# Traffic Congestion Detection System through Connected Vehicles and Big Data

**DOI:** 10.3390/s16050599

**Published:** 2016-04-28

**Authors:** Néstor Cárdenas-Benítez, Raúl Aquino-Santos, Pedro Magaña-Espinoza, José Aguilar-Velazco, Arthur Edwards-Block, Aldo Medina Cass

**Affiliations:** 1 School of Telematics, University of Colima, 333 Universidad Avenue, C.P. 28040 Colima, Col., Mexico; nestor_cardenas@ucol.mx (N.C.-B.); aquinor@ucol.mx (R.A.-S.); javelazco@ucol.mx (J.A.-V.); arted@ucol.mx (A.E.-B.); 2 Department of Innovation and Technological Development, Siteldi Solutions S.A. de C.V., 111 Canario Street, C.P. 28017 Colima, Col., Mexico; 3 Department of Innovation and Technological Development, Corporativo STR S.A. de C.V., 111 Canario Street, C.P. 28017 Colima, Col., Mexico; aldo.medina@corporativostr.com

**Keywords:** connected vehicles, big data, traffic congestion detection system, IoT

## Abstract

This article discusses the simulation and evaluation of a traffic congestion detection system which combines inter-vehicular communications, fixed roadside infrastructure and infrastructure-to-infrastructure connectivity and big data. The system discussed in this article permits drivers to identify traffic congestion and change their routes accordingly, thus reducing the total emissions of CO_2_ and decreasing travel time. This system monitors, processes and stores large amounts of data, which can detect traffic congestion in a precise way by means of a series of algorithms that reduces localized vehicular emission by rerouting vehicles. To simulate and evaluate the proposed system, a big data cluster was developed based on Cassandra, which was used in tandem with the OMNeT++ discreet event network simulator, coupled with the SUMO (Simulation of Urban MObility) traffic simulator and the Veins vehicular network framework. The results validate the efficiency of the traffic detection system and its positive impact in detecting, reporting and rerouting traffic when traffic events occur.

## 1. Introduction

Nowadays, the excessive population growth in urban settings represents one of the biggest challenges for governments worldwide. The rapid growth of city populations directly affects the ability of countries to reduce contamination in order to improve the quality of life of their citizens. The urban population in 2014 accounted for 54% of the global population, up from 34% in 1960 and is expected to grow approximately 1.84% per year between 2015 and 2020, 1.63% per year between 2020 and 2025 and 1.44% per year between 2022 and 2030 [1]. Caring for the needs of a rapidly growing urban population is a major challenge for countries trying to create more sustainable cities. A clear example of the impact of population growth on the environment is contamination occurring in the atmosphere, which increases proportionally to the number of people living in cities and the number of motor vehicles in circulation [2]. During the last 50 years, human activity, specifically the burning of fossil fuels, has released an indiscriminate amount of CO_2_ and other greenhouse gases, sufficient to retain more heat in the lower layers of the atmosphere thereby altering the global climate [3]. In addition to climatic change, air pollution also represents a major health hazard. According to a WHO report [4], air pollution in cities and rural areas throughout the world each year causes 3.7 million premature deaths, 80% of which are associated with diseases such as ischemic heart disease and stroke; 14% due to chronic obstructive pulmonary disease or acute infections of the lower respiratory tract and 6% to lung cancer. Globally, excessive air pollution is caused by unsustainable policies in sectors such as power generation, general industry and waste management, but mainly in the transportation sector [5]. Road vehicles are the major source of air pollution due to the use of fossil fuels, poor vehicle maintenance, old vehicles, poor fuel quality and the overuse of vehicles [6]. According to worldwide estimates published in 2014, there were a total of approximately 1.1 billion cars on the planet, which produced about 1,730,000,000 tons of CO_2_, a figure that is expected to grow over the next few years since some estimates predict that by 2035 the number of cars will reach close 2 billion [7]. In recent years a study published in Environmental Science & Technology reveals that local spikes in atmospheric CO_2_ concentrations may contribute to premature deaths by increasing ozone concentrations [8].

The excessive use of road vehicles not only harms both the environment but also affects the quality of life and the economy of the population. The phenomenon of traffic congestion is present in most developed cities. For example, according to the Dutch company Tom Tom [9], a worldwide company that produces navigation and mapping products using GPS technology and location-based products, the city of Istanbul, Turkey, is the world´s most congested city at 58%, followed by Mexico City and Rio de Janeiro, with 55% and 51%, respectively. In Mexico City, for example, commuters can spend as much as 23 h per month in congested traffic, a situation that not only exposes them to high contamination levels, but contributes significantly to stress and obesity problems [10]. The loss of prolonged time commuting and working hours also has a strong impact on the productivity of businesses and the overall economy. Annually, the traffic-generated economic losses amounts to over 1.5 billion dollars, or 1% Mexico City’s contribution to the country’s GDP, a very significant figure for a developing country [11].

This article discusses the simulation and evaluation of a traffic congestion detection system by means of connected vehicles and big data that accurately identifies traffic congestion, so that drivers can change their routes thereby reducing potentially harmful emissions and travel time. To meet the objective set, the developed system monitors, processes and stores large amounts of data reported from vehicles and fixed infrastructure, communicates to a big data cluster employing Location Routing Algorithm With Cluster-Based Flooding (LORA_CBF) which, through a series of algorithms, accurately identifies traffic congestion. The OMNeT++ discreet event network simulator was used to validate and evaluate the proposed system and the big data cluster [12]. The SUMO traffic simulator [13] was then coupled with the Veins vehicular network framework [14] to recreate a case study, based on a real traffic issue, to detect traffic congestion and the impact of the proposed system in terms of reducing traffic congestions.

## 2. Related Work

Today, the Internet is an indispensable tool for billions of people as it enables them to overcome many communication barriers (space, time, distance), as well as help them simplify many everyday activities. However, this was not always the case. In the past 50 years, the Internet’s evolution has been unprecedented as it evolved from being a small research network, consisting of a small number of nodes, to a huge global network that supplies services around the world [15]. It continues to grow at an incredible pace, expanding to other areas, devices and contexts of everyday life, due greatly to the advances and integration of Micro-Electro-Mechanical Systems (MEMS) and wireless communication technologies. These important advances have led to the development of increasingly smaller devices with monitoring, computing and wireless communications capabilities, all of which contribute to the Internet of Things (IoT) [16]. The relatively new discipline of ubiquitous computing, whose aim is to integrate technology into the activities or things used in everyday life, or to monitor the behavior of the world around us, was first conceived of in the late 1980s. Given the precedents of the Internet and ubiquitous computing, the IoT refers to the integration of devices with monitoring, actuation, computing and communication capabilities into everyday activities, using devices that can identify behavior in the physical world and use the infrastructure, tools and cyber space services to make decisions [17]. By 2020, it is estimated there will be more than 20 billion devices involved in the Internet of Things, in large part due to the expansion of the world’s telecommunications infrastructure, a figure 30% higher than the items/devices that currently exist [18]. With the growth of the Internet’s infrastructure, along with increased user growth and more integrated IoT devices, it is estimated that between 2005 and 2020, the volume of data worldwide will grow from 130 exabytes to 40,000 exabytes. Considering the challenges imposed by the increasing volume of data worldwide, the term “big data” was coined, which not only solves the paradigm of storing large amounts of data, which is seen as the new oil of the 21st century, but also considers processing schemes, using technologies and techniques to extract valuable and relevant information [19].

**Figure 1:**
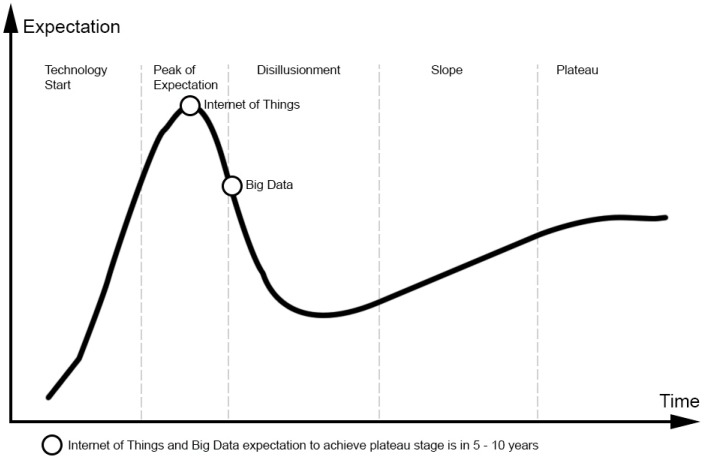
Big data and the IoT represented by hype cycle curve (source: elaborated by authors, based on [20]).

IoT and big data have been identified as two critical emerging technologies in the field of IT as shown in the IT Gartner Hype Cycle, shown in Figure 1. A Hype Cycle, is a study that can represent the novelty, adaptation, maturity and impact of specific technological applications. According to the study of emerging technologies [20], IoT and big data technologies will become technologies that are conventionally embedded in many everyday products in the next five to ten years.

Although large-scale adoption of big data technologies will take another 5 to 10 years, this time could be significantly reduced if big data challenges can be solved quickly. The complexity of data integration, real-time data handling, existing data warehouse architectures, data security and privacy represent some of the major challenges that restrict the adoption of big data technologies [21]. In response, several researchers have proposed new approaches and strategies based on scheme mapping, record linkage and data fusion, to reduce the complexity of data integration [22,23,24]. Additionally, some investigations have proposed novel architectures to process or analyze data in real-time big data applications [25,26,27]. Research along these lines is important as existing data warehouse technologies cannot currently handle the additional demands of new dynamic data sources and analytic workloads that users actually demand. For these reasons, tools are presently being developed to assist existing infrastructure migrate to new big data schemes [28]. Regarding data privacy and security challenges, several authors have addressed these topics and have implemented schemes and policies to encrypt and protect data source confidentiality [29,30,31,32]. Importantly, [33] proposes a novel approach ensuring privacy by preserving the centralized computation of moving objects. Additionally, the Liebig approach utilizes asymmetric cryptography using two keys in conjunction with Shamir’s secret. This cryptographic approach guarantees data security and preserves the data source privacy.

On the other hand, the IoT is presently the object of significant research as it will be implemented on a large scale within the next decade or so. Presently, several investigations have developed IoT applications for natural disasters [34,35], generalized and specialized industries [36,37,38], smart homes [39,40,41], medicine [42,43,44], agriculture [45,46,47], urban planning and intelligent cities [48,49,50] and the design of Intelligent Transport Systems (ITS) [51,52,53]. In the fields of ITS and smart cities, allowing vehicles to be interconnected, forming vehicular ad-hoc networks (VANETs), has enormous applications related to safety, internet connectivity, entertainment, file transfer and importantly, the detection and avoidance of dangerous or problematic traffic conditions [54]. Although VANETs have several applications, scalability and data aggregation represent two of their major challenges. Their large number of mobile nodes create a high volume of location querying and updating of messages, which often cause network congestion. Additionally, the large volume of data that each node shares with others causes a significant throughput decrease as the network adds new nodes, thus affecting network performance [55]. The authors of [55,56] concur that the end-to-end throughput available for each node tends to zero as the VANET grows, regardless of the routing protocol. In [55], Saleet affirms that data from multiple nodes can be combined using data aggregation techniques to ensure the scalability of protocols, even if the number of nodes significantly increases. Importantly, although data aggregation is often used in static Wireless Sensor Networks applications [57,58,59], they are increasingly being applied to VANETs [60,61,62]. Data aggregation techniques can be classified as either syntactic or semantic [62]. In the syntactic aggregation approach, vehicular data from several nodes are compressed into one package to reduce overhead. In semantic aggregation, the data from each node is summarized to report vehicular group location, forwarding smaller data packets, although with some accuracy loss.

Because traffic congestion represents one of the most important problems in urban environments, several researches have proposed possible solutions through VANETs and ITS. The AUTOPIA system, for example, proposes reducing traffic by implementing a control algorithm based on Fuzzy, which takes into account the information of each vehicle through vehicle-to-infrastructure communication (V2I) [63]. The authors of [64,65], propose helping solve traffic congestion problems by managing traffic lights, by altering the frequency and timing of the traffic light changes based on the number vehicles on each street. A video camera is used to detect vehicles and count them [66], an algorithm then detects the real-time traffic flow and possible traffic congestion. Other work carried out in [67] addresses the issue of vehicular traffic through prediction based information from cellular networks, proposing models to statistically correlate network information to incidents or events of vehicular traffic. In [68], the authors propose using a unique EPC (Electronic Product Code) to identify vehicles utilizing an RFID (Radio Frequency Identification) reader to read EPC code. Secondly, they obtain the position of vehicles by using GPS technology and then use GPRS technology to transmit the data of mobile objects to provide a vehicle count to detect congestion. Importantly, the significant work proposed in [69] provides a tool to extract information from social networks, particularly Twitter, related to traffic congestion in certain areas. The compilation of “tweets” is then processed by an algorithm to determine whether a specific area is congested or not. Exploring a different way to detect vehicular traffic, this paper promotes the use of vehicle-to-vehicle (V2V), vehicle to infrastructure and infrastructure-to-infrastructure (I2I) communication to enable vehicles to share their positions, speeds, paths and travel times. With that data, a big data cluster can identify traffic congestion events and inform drivers so that they can make real-time decisions to avoid traffic congestion. Importantly, the big data cluster generates metrics that can lead to public policies for cities to improve the quality of life of its habitants from the environmental, health and economic points of view.

## 3. Proposal Architecture

### 3.1. Architecture

**Figure 2:**
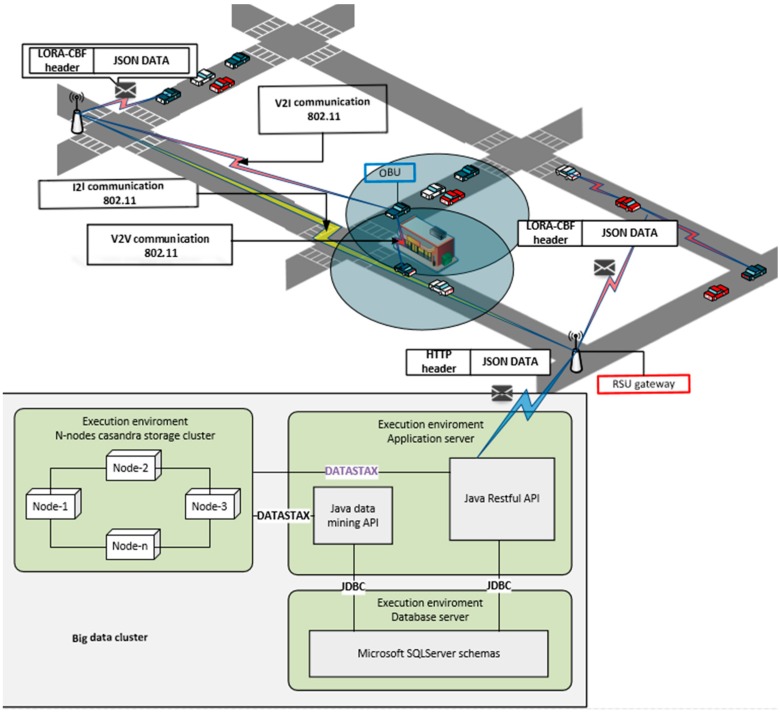
General architecture of the traffic detection system.

This section explains the proposed architecture for the system. Figure 2 shows the general traffic detection system. The proposed system includes the use of vehicular communication and big data clusters to detect traffic congestion using V2V, V2I and Infrastructure to Infrastructure (I2I) communications. The system uses the LORA-CBF algorithm because it has been previously validated in urban scenarios. LORA-CBF was selected because it is a dynamic, hierarchical (gateway, cluster heads and member roles), multi-hop algorithm, which avoids the flooding of control packets and controls unnecessary retransmissions [70,71,72]. To achieve V2V and V2I communication, the system uses On-Board Units (OBU) which obtain vehicle position and speed at one-second intervals and forwards their data to cluster head cars, which are responsible for performing the data aggregation, an approach reported in [73]. To forward the aggregated data, cluster head cars send the information to the Road Side Unit (RSU) directly or through cluster/gateway nodes. The RSUs are then responsible for forwarding the information packets to the big data cluster for storage and further processing to determine if significant traffic congestion is present in specific locations. All the vehicular information is encapsulated in DATA packets by the LORA-CBF algorithm which permits the network nodes to both transmit and receive them. When DATA packets are received by an RSU, they are un-encapsulated and re-encapsulated by Hypertext Transfer Protocol (HTTP) for their transmission in a TCP packet to the big data cluster.

### 3.2. Big Data Cluster

Cars forming part of the network of connected vehicles generate a continuous stream of information that is sent to the Cluster which receives it by means of an API resident on an application server, responsible for receiving and interpreting data in order to detect traffic congestion events.

This section details the features of the Cluster and defines criteria for interpreting traffic congestion events.

Traffic congestion is considered any event which combines low vehicle speeds and long queues formed by this slow moving cars. The system maintains a registry with the threshold speed and length of acceptable cues for each road in the scenario to enhance the sensibility of the traffic congestion alert generation.

The cluster is composed of 10 identical computers and an application server. Each one of the computers possesses 16 GB DDR3L RAM, 500 GB HDD, Core i7 processor at 2.2 GHz, and a Gigabit Ethernet interface. The 10 computers are connected in a star topology to a 10/100/1000 Base-T 24-port Switch. Each computer runs a 64-bit Ubuntu 14.04.1 LTS operating system, to which Java 1.7.0_75 and Cassandra 2.0.2 were installed. The application server has 16 GB DDR3 RAM, 1 Tb HDD, Intel Xeon E5 processor at 2.4 GHz and a Gigabit Ethernet interface, running a Windows Server 2008 operative system, to which SQL Server 2008 R2, JDK—JRE 1.8 and Tomcat 8 were installed.

**Figure 3:**
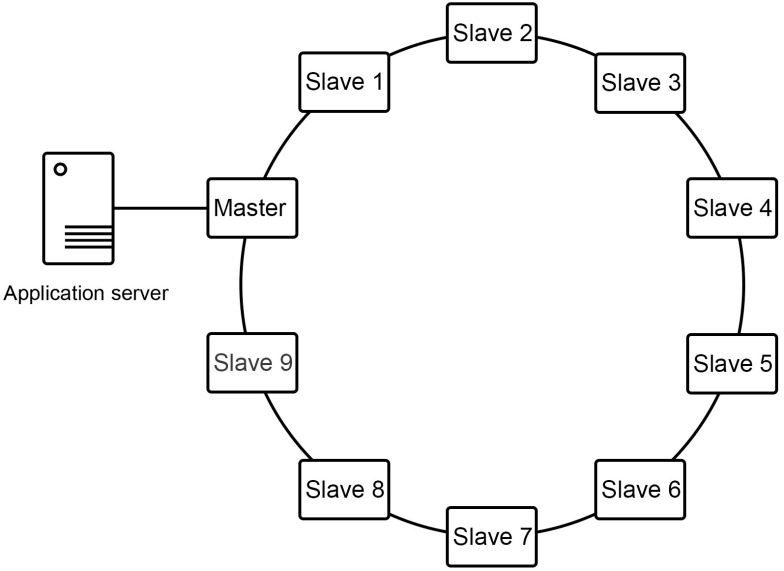
Diagram of the big data cluster network.

The cluster is formed by a node that serves as Master and 9 nodes that act as slaves. All nodes are configured in a communication ring to selectively share their public keys, allowing each node to connect via SSH with its two neighboring nodes without a password. The application server communicates only with the master node using SSH, as shown in Figure 3.

**Figure 4:**
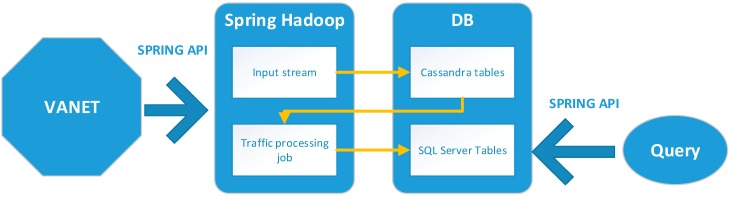
Big data architecture.

For this application, we decided to use a Kappa architecture because the system does not require the speed layer of the lambda architecture to process the real-time data. The badge collects the incoming stream in a specified amount of time to estimate traffic congestion events. Figure 4 shows the used architecture and the main data flow.

**Figure 5:**
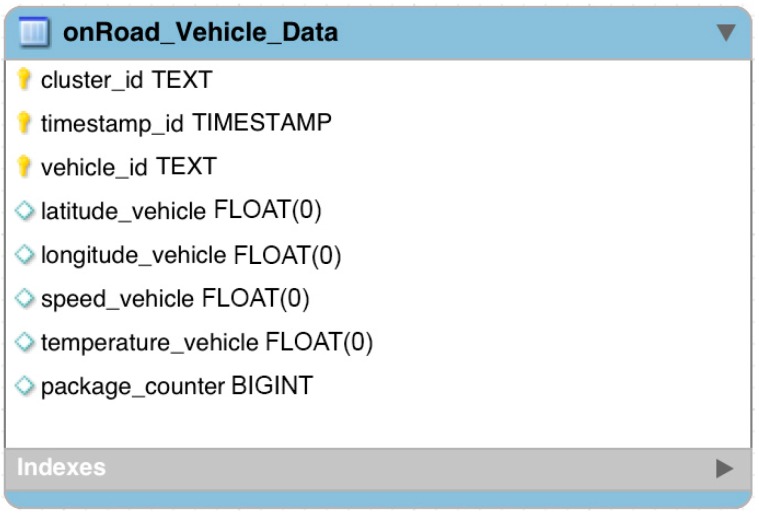
Cassandra data model to store vehicular information.

Cassandra is used to support a high flow of inserts and queries, using its distributed architecture. Figure 5 provides the data model implemented in Cassandra to store and view vehicle information:

The OnRoad_Vehicle_Data model consists of an integrated primary key, which is composed by cluster_id to identify each of the monitoring points, timestamp id to provide the date and time when the vehicular information was sent and a vehicle id to provide a unique identifier for each car in the VANET. The data in each record includes latitude vehicle, longitude vehicle, speed vehicle, temperature vehicle, as well as a package counter. All of this information is sent at one-second intervals from the vehicles in the VANET to the application server which forwards all the information to the Cluster.

**Figure 6:**
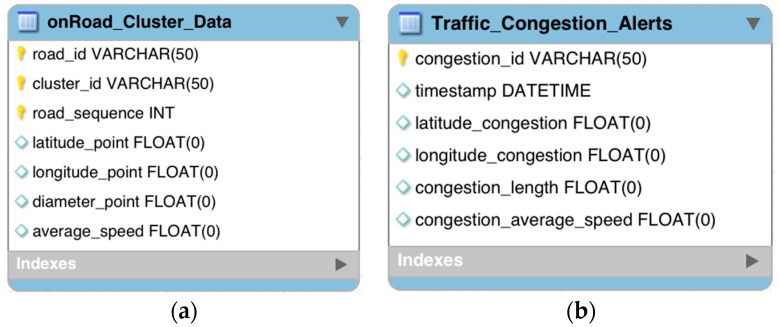
(**a**) Data model to store catalog coverage points; (**b**) data model to store the detected alerts.

SQL Server is used to store a traffic event log from the information contained in Cassandra, as well as a catalog of coverage points. Figure 6 shows how the model is implemented:

The OnRoad_Cluster_Data model is a catalog containing the following fields: road_id to provide the street on which the vehicles are traveling; cluster_id to define the coverage points; road_sequence to identify a number of consecutive coverage points on the street, applicable in settings where the streets are linear or curved; latitude_point and longitude_point to determine the decimal format GPS coordinates of the center point of the coverage; diameter_point to determine the coverage diameter in meters from the point of coverage and average_speed to provide the average speed in km/h for cars with the diameter of the coverage point.

The Traffic_Congestion_Alerts model is a set of traffic alerts which contain a primary key called congestion_id, a unique alert identifier, the timestamp of the alert, latitude_congestion and longitude_congestion which keeps the approximate traffic congestion epicenter. Congestion_length shows the approximate traffic congestion length in meters and the average_speed field represents the average speed of cars within the traffic congestion.

The application server runs a RESTful API using the Spring framework. This application has two primary jobs: (1) to receive information from the cars in the VANET; (2) to send the alerts to the cars that consult it. The RESTful API receives information and forwards alerts by means of the following functions:

The **Insert** function receives a series of parameters in JSON format and saves them in the Cassandra cluster. The parameters received include the following:

- Acknowledgement: a 0 (false) or 1 (true) condition that states whether or not a vehicle has been previously recognized.
- Latitude: the latitude in a decimal format.
- Longitude: the longitude in decimal format.
- Temperature: a floating number that indicates temperature.
- Vehicleid: a text chain that identifies the vehicle.
- VehicleIp: an IP address that identifies a vehicle in the network.
- packageCounter: a whole number that indicates the number of the received package.
- Speed: a floating number that indicates the speed of the vehicle.
- Timestamp: the time and date in which the registry was sent.

The **Request** function sends alerts of existing traffic, according to the positional time and radius parameters. These parameters are sent in JSON format with the following structure:

- Latitude: the latitude in decimal format.
- Longitude: the longitude in decimal format.
- Vehicleid: a text chain that identifies the vehicle.
- VehicleIp: an IP address that identifies a vehicle in the network.
- Timestamp: the time and date in which the consult was sent.
- congestionSearchRadius: the radius in meters in which the registered traffic will be searched.
- congestionDetecionTimeframe: the time in seconds to consider previous alerts.

After sending set of data, the API responds by providing a list of traffic events within the specified time and radius. Each traffic event contains the following data:

- vehicleId: a text chain that identifies the vehicle that filed the request.
- vehicleIp: an IP address that identifies the vehicle that filed the request.
- congestionAlertId: a unique identifier of the alert.
- timestamp: the time and date in which the traffic event was detected.
- Latitude: the approximate latitude from the central point of traffic in decimal format.
- Longitude: the approximate longitude from the central point of traffic in a decimal format.
- congestionLength: the longitude of traffic in meters.
- congestionAvgSpeed: the average speed of circulation in km/h.

**Figure 7:**
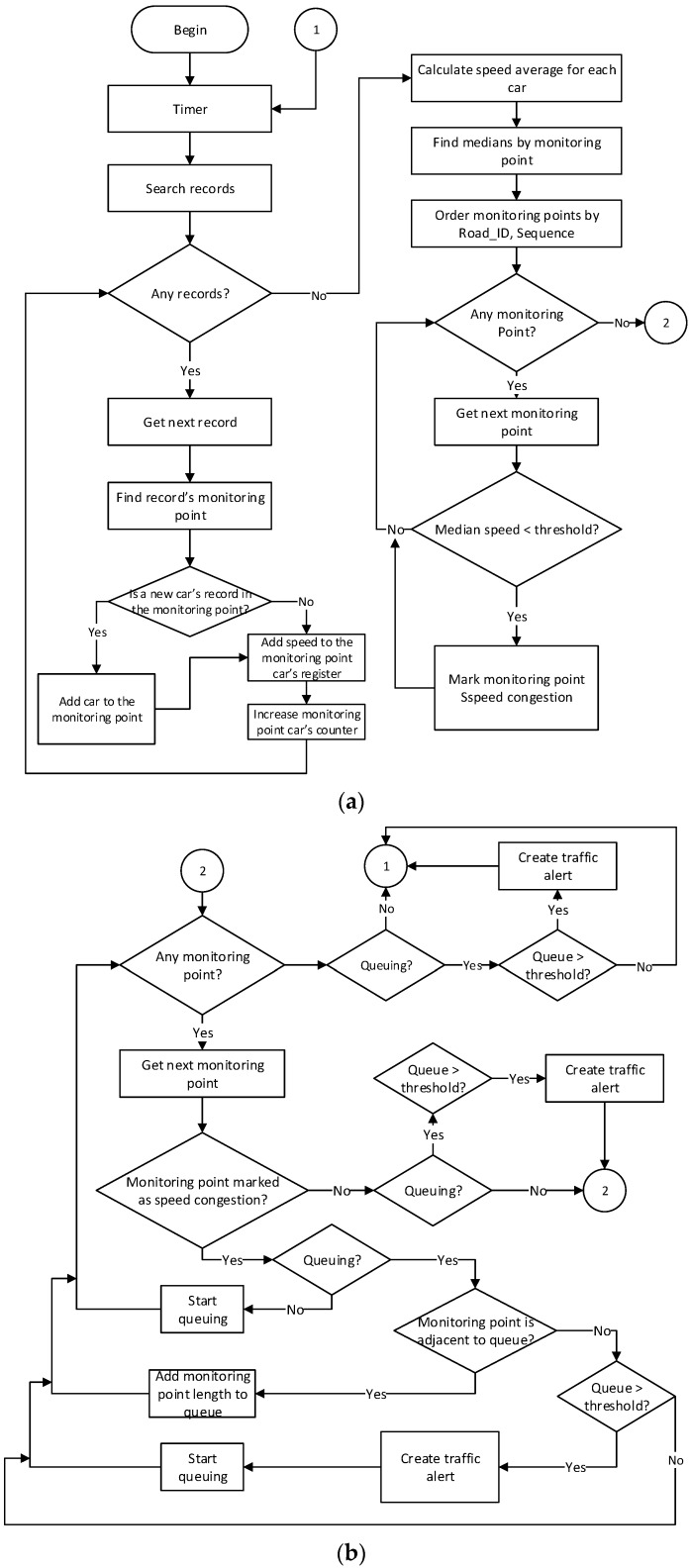
(**a**) Traffic event detection algorithm part 1; (**b**) Traffic event detection algorithm part 2.

Cassandra tables are consulted by the Spring Hadoop job to generate a traffic alert every 5 minutes, employing data received during the previous 5-minute period. All records are processed according to the algorithm shown in Figure 7, which identifies traffic events and stores them in the table Traffic_congestion_Alerts SQL Server.

The traffic event detection algorithm shown in Figure 7 is based on the BTO algorithm proposed by [74]. The algorithm begins by loading the initial configuration settings which include the time between iterations, speed thresholds, length thresholds and filter time. After this, the process timer employs the preset configuration parameter values as its input, initiates the values to be used and waits for the specified standby time before continuing. Following this, a query is made to the OnRoad_Vehicle_Data model, whose motor filters the records created during the previously assigned period of time. The query’s results are subsequently used to obtain individual records.

The algorithm then consults the database to discover the coverage point each record belongs to. This process consists of calculating the distance, using Equation (1), between the point contained in each vehicle’s record and the entire group of the monitoring points. A vehicle’s record corresponds to a monitoring point when the distance between the points is equal to or less to than the radius of the monitoring point.

(1) $$d=2r\text{ arcsin}\left(\sqrt{sin^{2}\left(\frac{\Phi_{1}-\Phi_{2}}{2}\right)+cos\left(\Phi_{1}\right)\;cos\left(\Phi_{2}\right)\;sin^{2}\left(\frac{\lambda_{2}-\lambda_{1}}{2}\right)}\right)$$

Equation (1) shows the haversine formula used to calculate the distance between two points, where Φ_1_, Φ_2_ are the latitudes of the two given points and λ_1_, λ_2_ are their corresponding longitudes.

After discovering the monitoring point for the record, the algorithm summarizes its speed with the rest of the record’s speeds belonging to the same vehicle. Once all of the registers are processed, the average speed of each vehicle is calculated at each monitoring point. Following this, the algorithm searches for the median speed of each monitoring point, which requires the average speed of a minimum of three vehicles to provide reliable alerts for a real-time scenario. Next, the algorithm orders the monitoring points using the Road_ID and Sequence numbers to produce a list that is used as the cycle input, in which the monitoring points are marked as “Speed congested” only when the median speed is slower than the minimum speed threshold. Once all of the monitoring points are processed, they pass to a second cycle where each is evaluated to find the sequences of monitoring points that are marked as speed congested, which are considered queues. The queue length is given by the sum of the diameters of the monitoring points that comprise the queue. If the queue length is longer than the given threshold for the road where the queue is in, a traffic congestion alert is created, including the traffic line length and the central point of the traffic congestion. The algorithm then saves the traffic congestion alerts into the Traffic_congestion_Alerts model. Finally, the algorithm returns to the timer.

### 3.3. On-Board Unit

The OBU device installed in the vehicles possess a System on Module (SoM) that includes a Cortex A9 quad core processor at 1 GHz with 1 GB DDR3 of RAM memory on a 64 bit architecture. Also, the OBU possesses a touch-screen and an 802.11 b radio which supports ad-hoc communication with a transmission capacity of 12 dBm and an omnidirectional antenna with a 5 dBi gain. The OBU performs the LORA-CBF communication protocol and a monitoring algorithm to propose alternate routes.

#### 3.3.1. Route Monitoring Algorithm

**Figure 8:**
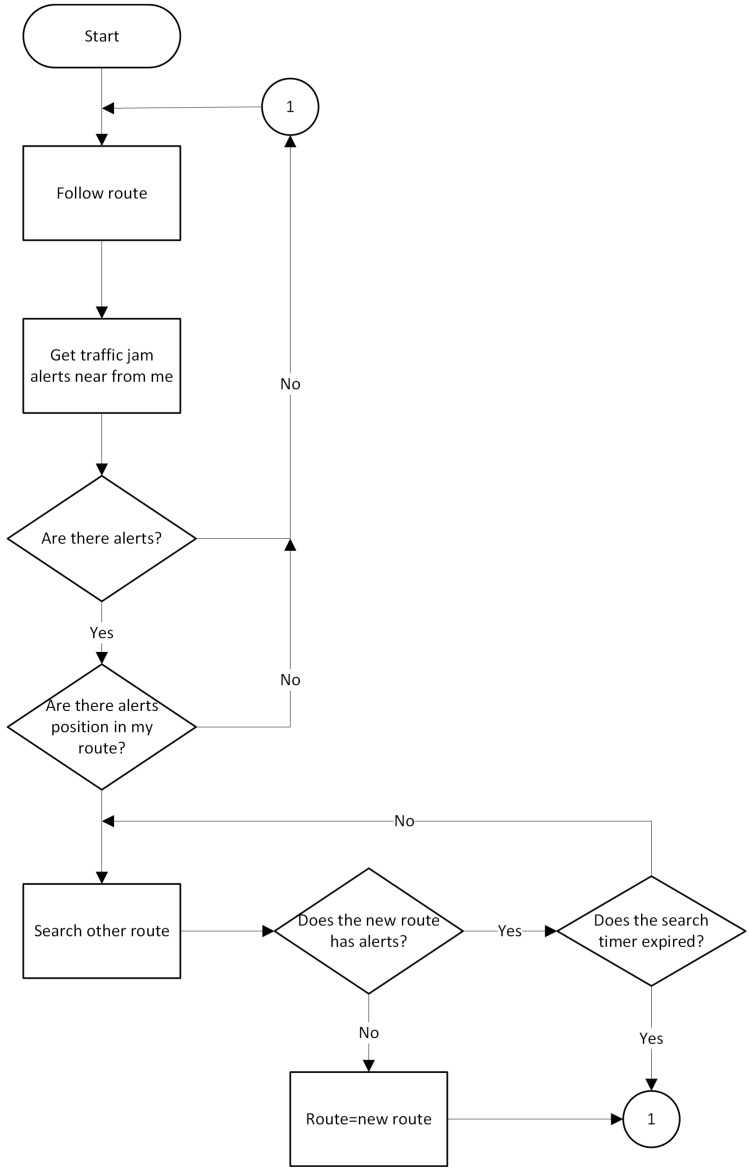
Route Monitoring Algorithm.

The route monitoring algorithm is responsible for observing the path where the car should be free from traffic at one-minute intervals. If there is a traffic congestion alert near the car or if any alert is on the current vehicle route, the algorithm seeks and assigns a new path which contains no traffic congestion alerts. If the process of finding a new alert-free route does not succeed, or the search time expires, the same route is maintained. The route monitoring algorithm shown in Figure 8.

#### 3.3.2. Algorithm LORA-CBF

**Figure 9:**
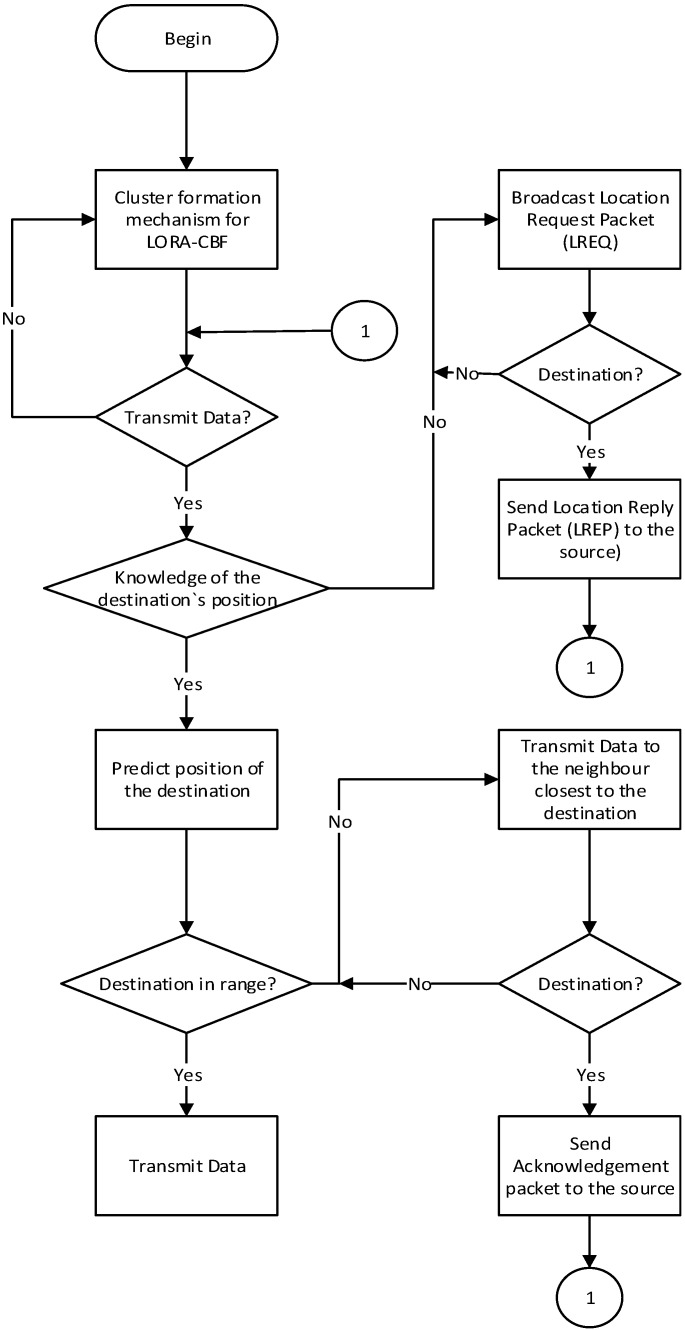
LORA-CBF communication algorithm.

The LORA-CBF communication algorithm works hierarchically. It is formed by a cluster head, zero or more members in each group and one of more link portals to communicate with the other cluster heads. Each cluster head contains a “Cluster Table”. A “Cluster Table” is defined as a table that contains the address and geographical location of the members and Gateway nodes. The cluster-forming mechanism is the first to be executed and is maintained at all times as shown in Figure 9.

When a source tries to send data to a destination, it first checks the routing table to determine if the destination’s location is known. If it is, it sends the packet to the neighbor closest to the destination. Otherwise, the sources stores the data in its intermediate memory, starts a timer and Location Request (LREQ) packet transmissions. Only Gateway nodes and cluster heads can transmit a LREQ packet.

Gateway nodes only transmit gateway packets to one another in order to minimize unnecessary transmissions and only if the gateways belongs to different cluster groups. By receiving a request of location, each cluster confirms that the destination is a member of its cluster. If successful, it generates a Location Reply (LREP) packet that returns to the sender using geographical routing since each node knows the location of the source and the closest neighboring node, based on the information of the received LREQ packet.

Once the source receives the destination’s location, it recovers the data packet from its memory and sends it to the neighbor closest to the destination.

**Figure 10:**
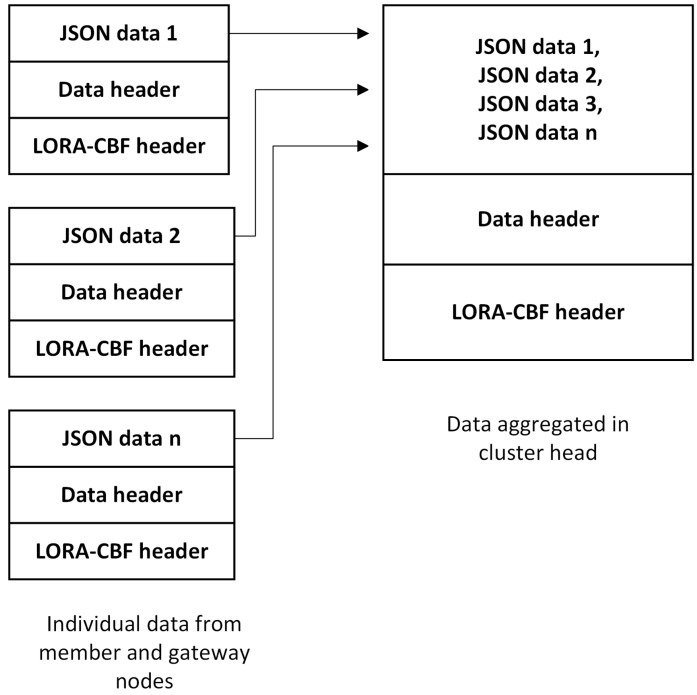
Construction of the syntactically aggregated data.

The location and speed data of each node are inserted in the big data cluster every second as this type of data is the most abundant in the network. For this reason, it is necessary to implement a data aggregation strategy to not affect network performance as the network grows. Data aggregation is performed using a syntactic technique which is applied by the cluster head to all of the data received from its members each second. Figure 10 illustrates the data aggregation process performed by the cluster head.

If a node functions as the gateway role, it will choose only one cluster head to which to send its data, based on the distance and will notify other cluster heads that it will not send its data to them. In order to archive better network performance with a significantly greater number of vehicle nodes, location and speed data inserts of each node do not have to receive a driver acknowledgement.

### 3.4. Road-Side Unit

The RSU device is responsible for communicating vehicles with the fixed infrastructure and forwards the information to a Gateway before resending the data compiled from the vehicular network to the big data Cluster. The RSU can function as a gateway when it connects to another infrastructure. It is comprised of a System on Module (SoM) that contains a Quad Core Cortex A9 processor at 1 GHz and a 1 GB DDR3 64 bit RAM memory. Additionally, the RSU has an 802.11 b radio that supports ad-hoc communication with 12 dBm transmission power and an omnidirectional antenna with 9 dBi gain for V2I and I2I communication. When the RSU functions as a Gateway, it will connect either via Ethernet to the same network segment as the big data Cluster, or directly to the internet to communicate to the big data Cluster.

### 3.5. Design of the Packet Structure

The design of the packet structure used in both the OBU and the RSU to insure the proper flow of LORA-CBF, as well as the message capsule used by the big data Cluster to detect traffic, are described in the following tables.

Table 1 shows the header and structure of protocol packets, which are encapsulated in a UDP datagram. This type of packet forms the basic structure for all the packets handled by the protocol.

- **Begin**: a special character to indicate the beginning of the packet.
- **Length**: the number of bytes contained between the size data and the verification sum.
- **Transmission method**: the manner in which the packet is sent (Multicast, Broadcast, Unicast).
- **Source Address**: the address of the sending node (changes with each jump).
- **Destination Address**: the address of the receiving code (changes with each jump).
- **RSSI**: the received signal intensity (dBm).
- **Payload**: the content of the packet with a maximum size of 1488 bytes.
- **Checksum**: a number for checking the packet’s integrity.
- **Forcer:** a byte to force the 802.11 transceptor to send the datagram and it doesn’t wait to fill its maximum capacity.

#### 3.5.1. Types of Packet Payload Content

The following tables provide the different packet structures within payload, depending on their types. A brief explanation of each field is also shown.

Table 2 shows the structure of a HELLO packet used to discover new neighbors and to create or change clusters according to the cluster forming mechanism shown in Figure 9.

The fields for the HELLO packet include:

- **Packet type:** a code corresponding to the packet type.
- **Node type:** a code corresponding to the node type (cluster head, member, gateway).
- **Latitude:** the 12-byte latitude given by the GPS.
- **Longitude:** the 12-byte longitude given by the GPS.
- **Speed:** the vehicle speed.

Table 3 shows the structure of a LREQ packet used when the origin of a DATA packet is transmitted to a destination that is not included in its routing table, or when the route has expired. The identification field of an LREQ packet allows it to determine whether or not an LREQ packet has been seen previously. If an LREQ packet has been previously seen, subsequent LREQ packets will be discarded.

The fields for the LREQ package include:

- **Packet type:** a code corresponding to the packet type.
- **Identification field:** the field that records if a packet has been previously seen.
- **Node type:** a code corresponding to the node type (Cluster Head, Member, Gateway)
- **Applicant address:** the address of the node that initiates the search.
- **Address to search: the** node address to be discovered.
- **Latitude:** the 12-byte latitude given by the GPS.
- **Longitude:** the 12-byte longitude given by the GPS.
- **Speed:** the vehicle speed.

Table 4 shows the structure of a LREP packet used by the cluster head to respond to the source node if the destination node is a member of the cluster. It is sent using a unicast transmission method.

The fields for the LREP package include:

- **Packet type:** a code corresponding to the packet type.
- **Node type:** a code corresponding to the node type (Cluster Head, Member, Gateway).
- **Applicant address:** the address of the node initiating the search.
- **Address to search:** the node address to be searched.
- **Latitude:** the 12-byte latitude given by the GPS.
- **Longitude:** the 12-byte longitude given by the GPS.
- **Speed:** the vehicle speed.

DATA, DATA REQUEST and DATA RESPONSE packets contain all the information obtained from the different devices within the network. Table 5 shows the structure of a DATA packet used to transmit any information to the vehicular network and the server.

Table 6 shows a REQUEST packet used to send a petition to the server. This type of packet waits for a response from the server.

Table 7 shows the structure of a DATA RESPONSE packet used to respond to the network device that requests data from the server through the Gateway.

The fields for the DATA, DATA REQUEST and DATA RESPONSE packages are explained below:

- **Packet type:** a code corresponding to the type of DATA.
- **Initial source address:** the origin address of the maintained data.
- **Final destination address:** the destination direction of the maintained data.
- **Hops:** the number of jumps from the data’s origin.
- **Packet counter:** an incremental packet counter of the data source.
- **Data:** the information that will be sent through the network.
- **Latitude:** the 12-byte latitude given by the GPS.
- **Longitude:** the 12-byte longitude given by the GPS.

#### 3.5.2. DATA Packet De-Encapsulation and Encapsulation

DATA, DATA REQUEST and DATA RESPONSE packets received by a RSU with a Gateway functionality will be de-encapsulated to obtain the data, which is then sent by means of an HTTP protocol to the API of the cluster in a JSON format. The vehicle data and the big data Cluster that uses the vehicular data are then encapsulated in packets as shown in Table 8.

The data delivered by the server is encapsulated by the link port RSU and then sent to the OBU that requested it.

## 4. Evaluation

### 4.1. Evaluation Scenario

**Figure 11:**
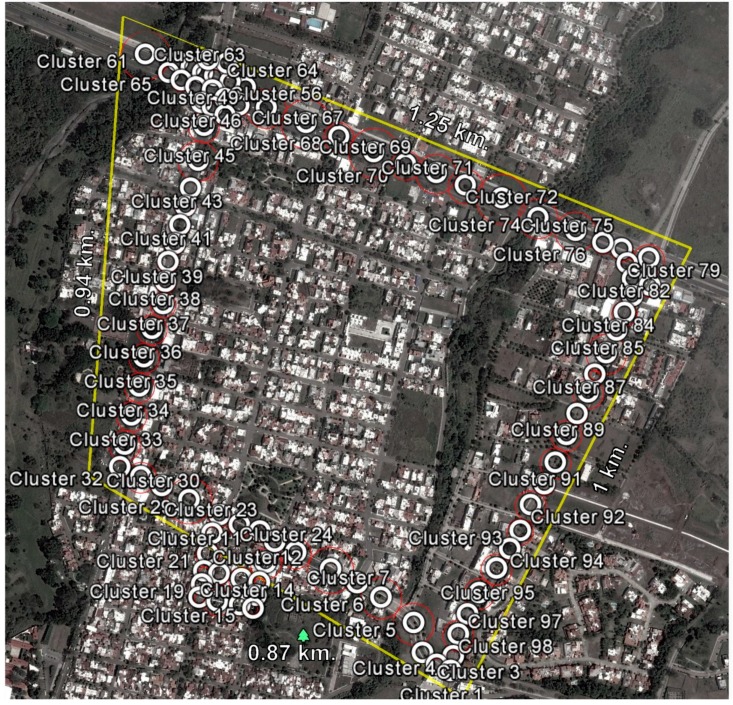
Evaluation scenario with the coverage points in place.

The scenario for the evaluation of the traffic detection system is shown in Figure 11, which includes important streets in the city of Colima, Mexico that presents significant traffic congestion during peak hours, mainly at street intersections.

**Figure 12:**
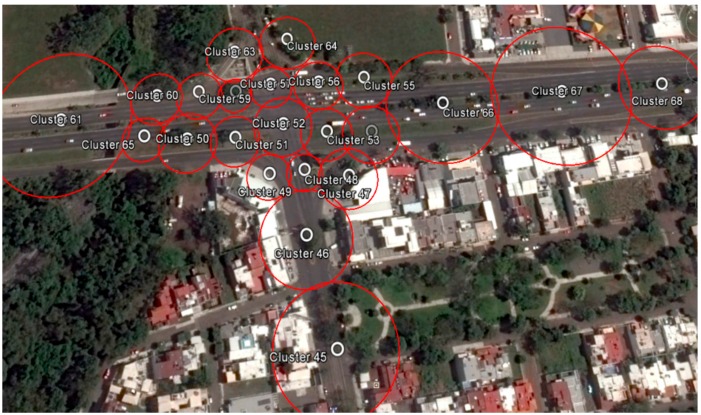
Detailed evaluation scenario.

The scenario shown in Figure 11 was previously studied in order to establish monitoring points along the avenues and at the main intersections of the study area. Each monitoring point was geographically referenced with its specific monitoring diameter and information concerning the average speed of its area of coverage, data which was required by the traffic detection algorithm. It is important to note that it was necessary to add more monitoring points at the main street intersections in order to avoid false alerts in areas where stoplights exist, as shown in Figure 12.

### 4.2. Simulation Model

**Figure 13:**
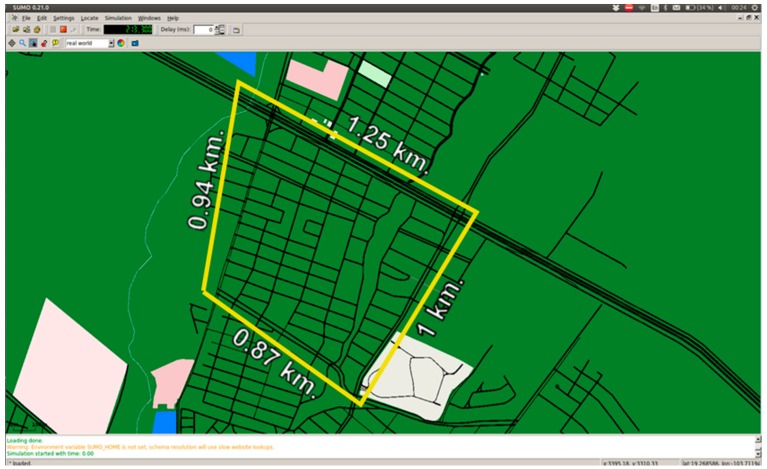
Complete scenario programmed in SUMO.

**Figure 14:**
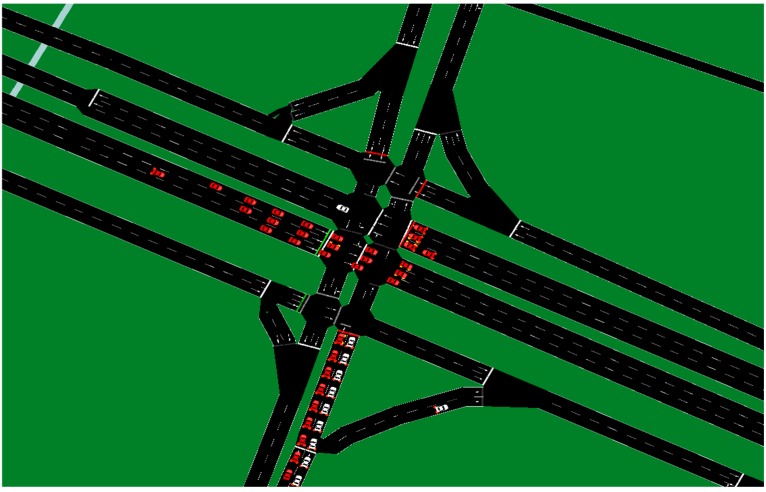
Amplification of the intersection scenario programmed in SUMO.

To validate the traffic detection algorithm, the vehicular flow was simulated in the proposed evaluation scenario using SUMO, in which the corresponding vehicular flows during peak times were programmed. Additionally, the traffic light intervals were also programmed in the SUMO simulation. The programmed scenario is shown in Figure 13 and Figure 14.

Because of the large number of vehicles in circulation during peak hours, congestion is often generated at traffic lights. Consequently, multiple traffic congestion was generated to better validate the traffic detecting algorithm using the OMNET++ simulator, coupled with the SUMO mobility simulator and the Veins framework. The generated traffic congestion was programmed using a range of different vehicles at different points of the scenario. In this way, we could observe the behavior of the algorithm and count the number of traffic alerts generated in relation to the number that were programmed, as well as false alerts attributable to the number of vehicle with an OBU. A total of 236 vehicles were used in the simulation because *in situ* observations established this was on average, the number of cars causing traffic congestion in the real-world study area. In the first test, 118 of the vehicles had an OBU, represent 50% of the total of vehicles. In the second test, only 24 vehicles possessed an OBU, representing 10.1% of the vehicle total.

Vehicle connectivity was programmed using the Veins Framework, the OMNET ++ discrete event simulator and the LORA-CBF algorithm, as shown in Figure 9. The above-mentioned framework was used to simulate the route monitoring algorithm proposed in Figure 8. Each vehicle was assigned a pre-established route assessed by the route monitoring algorithm to define route changes, depending on the results of the traffic congestion detection algorithm proposed in Figure 7. If congestion was present along the route, the route monitor algorithm using the Veins Framework informed the SUMO mobility simulator it needed to change the route, allowing vehicles to avoid congestion detected by the traffic detection algorithm.

**Figure 15:**
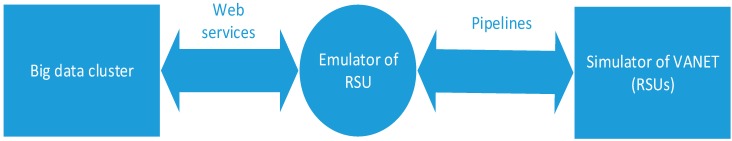
Operation diagram of the RSU emulator.

To validate the traffic detection algorithm sent to the big data cluster, it must first communicate vehicle data provided by the simulation in real time. This program was generated in C++ and emulates part of the operation of the RSU, which is responsible for communicating the big data cluster and RSU simulator and functions as a gateway between the simulators and the big data cluster. To achieve RSU transmission of data sent to the big data cluster, we developed a communication channel between the simulation and the big data cluster through UNIX pipes. The communication channel sends and receives bi-directional information from both the vehicles and the big data cluster, sending and receiving information from the RSU emulator through web services. Following this, the RSU inputs the data received from the vehicles and consults the traffic alert information every 4 minutes. Figure 15 shows the conceptual operation of the RSU emulator.

### 4.3. Cluster Configuration

**Figure 16:**
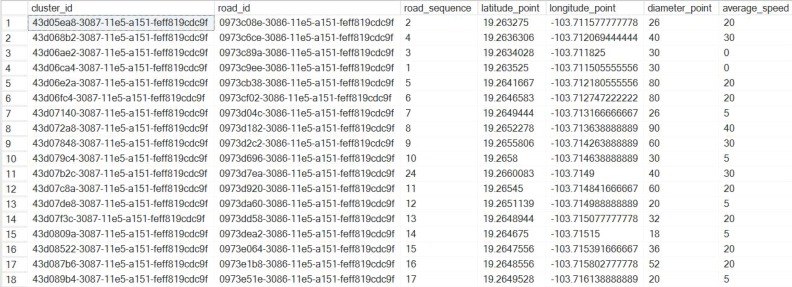
Records contained in the OnRoad_Cluster_Data model.

The cluster was configured to evaluate the scenario provided in Figure 11. Ninety-eight coverage points were input into the OnRoad_Cluster_Data model. Of these, 98 corresponded to the evaluation scenario and a single registry corresponded to an undefined cluster, to which new registers unrelated to the already existing clusters will attach. Figure 16 provides an example of some of the registries corresponding to coverage points.

The iteration time for the big data cluster to analyze the data stored was set at 5 min. This 5-minute interval was determined after simulations using different time intervals, concluding 5 min is sufficient time to obtain representative data of traffic events.

## 5. Results

### 5.1. Performance of the Traffic Congestion Detection System

The results were obtained by simulating 64 traffic congestions in different avenues and streets and at different times. Table 9 shows the results of two tests where the top row contains the first test’s results and the lower row contains the second test’s results. The first test results indicate that 63 alerts were generated by the big data cluster, with one false alerts and two alerts which were not generated. The first test presented a traffic detection accuracy of 93.7% in a scenario with a total of 236 vehicles, of which 24 (10.1%) had OBUs.

The second test results indicated that the big data cluster generated 63 alerts, with zero false alert and one traffic congestion alert which was not generated. The second test presented a 98.4% accuracy in detecting traffic congestion in a scenario in which 118 vehicles, or 50% of all vehicles, had an OBU.

The results of both tests show that the accuracy increases when more vehicles use OBU, although the accuracy percentage remains acceptable when a smaller number of vehicles use OBU.

### 5.2. Time and CO_2_ Reductions Achieved by the System

**Figure 17:**
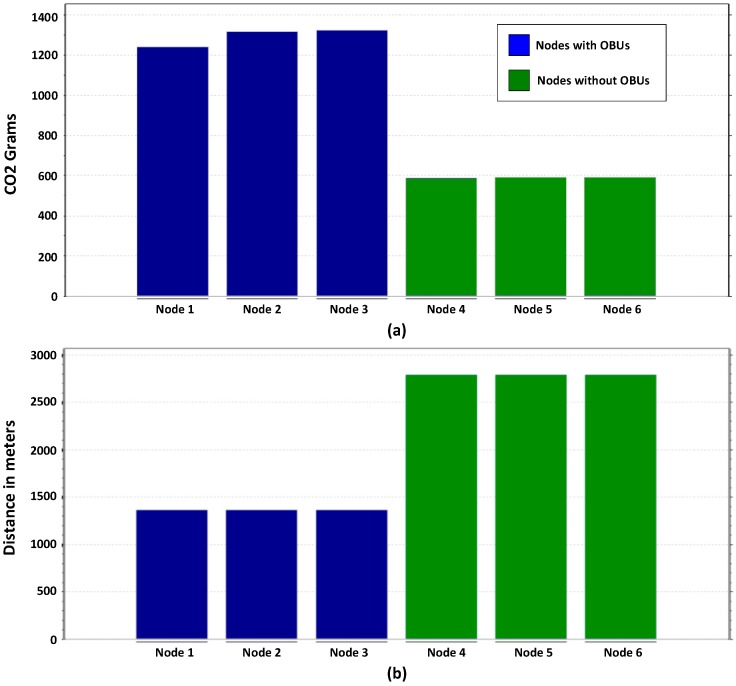
Comparison of the simulation results: (**a**) Comparison of CO_2_ emissions; (**b**) Comparison the distance traveled.

**Figure 18:**
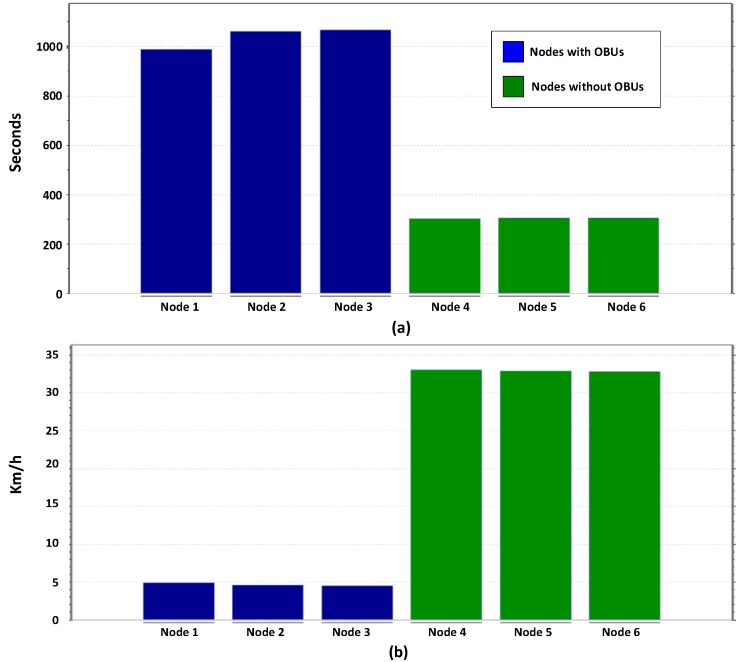
Comparison of the simulation results: (**a**) Comparison of the time traveled; (**b**) Comparison of the average speed of vehicles.

The results obtained are shown in Figure 17 and Figure 18 show a reduction in the time required to reach a destination in vehicles using the OBU module (Nodes 4, 5 and 7). Data also shows a significant reduction in carbon dioxide emissions of vehicles receiving traffic congestion alerts by changing their route. By increasing the average vehicle speed and reducing congestion, CO_2_ emissions are significantly reduced when compared to the emissions of congested cars idling that do not use the OBU module (nodes 1–3). The formula used to calculate the CO_2_ generated by vehicles is provided by [75].

## 6. Conclusions

This paper has presented a traffic congestion detection system that communicates connected vehicles and big data, to improve traffic flow and reduce CO_2_ emissions in the city, representing a potentially valuable tool to help drivers detect and avoid vehicular congestion.

The evaluation of the presented system allows us to conclude:

- The algorithm’s accuracy in detecting traffic congestion with 10.1% of the vehicles equipped with an OBU is of 93.7% in 64 programmed traffic congestions.
- The algorithm’s accuracy in the detection of traffic congestion of 50% of the vehicles with an OBU in place, increases to 98.4% in 64 programmed traffic congestions.
- CO_2_ greenhouse gas emissions are reduced by 50% on average, by detecting and conveniently modifying the route.
- The average arrival time to the destination is 70% shorter, by detecting traffic congestion and changing the routes.

Future work includes other simulation scenarios to improve the system and implement the OBU and RSU devices on-the-fly in real-world scenarios, to detect traffic congestion and reduce contaminant emissions. In the future, the RSU device will include the detection of ozone (O_3_), sulphur dioxide (SO_2_), nitrogen dioxide (NO_2_), carbon monoxide (CO), nitrogen dioxide (NO_2_), 2.5 (PM_2.5_) and 10 (PM_10_) particles, which are more harmful to persons living in urban settings. Additionally, for real-world implementation, the system will include a novel mechanism for vehicular localization in urban canyons, search for situation aware routes and a new approach to implement data security and privacy. Additionally, the RSU wireless trunk will be used to support other applications or services for smart cities.

## Figures and Tables

**Table 1:** LORA-CBF Packet Structure.

Begin	Lenght		Type Send	RSSI	Reserved	Source Address	Destination Address	Payload	Checksum	Forcer
7E	00	08	01	1 Byte	1 Byte	4 Bytes	4 Bytes	0–1488 Bytes	9D	7D

**Table 2:** LORA-CBF HELLO Packet Structure.

Packet Type	Node Type	Latitude	Longitude	Speed
48	01	12 Bytes	12 Bytes	1F

**Table 3:** LORA-CBF LREQ packet structure.

Packet Type	Identification Field	Node Type	Applicant Address	Address to Search	Latitude	Longitude	Speed
68	01	01	4 Bytes	4 Bytes	12 Bytes	12 Bytes	1F

**Table 4:** LORA-CBF LREP packet structure.

Packet Type	Node Type	Applicant Address	Address to Search	Latitude	Longitude	Speed
78	01	4 Bytes	4 Bytes	12 Bytes	12 Bytes	1F

**Table 5:** LORA-CBF DATA packet structure.

Packet Type	Initial Source Address	Final Destination Address	Hops	Packet Counter	Data	Latitude	Longitude
44	4 Bytes	4 Bytes	02	3 Bytes	0–1455 bytes	12 bytes	12 bytes

**Table 6:** LORA-CBF DATA REQUEST packet structure.

Packet Type	Initial Source Address	Final Destination Address	Hops	Packet Counter	Data	Latitude	Longitude
45	4 Bytes	4 Bytes	02	3 Bytes	0–1455 Bytes	12 Bytes	12 Bytes

**Table 7:** LORA-CBF DATA RESPONSE packet structure.

Packet Type	Initial Source Address	Final Destination Address	Hops	Packet Counter	Data	Latitude	Longitude
46	4 Bytes	4 Bytes	02	3 Bytes	0–1455 Bytes	12 Bytes	12 Bytes

**Table 8:** Example of an encapsulated DATA packet.

**Field**	**Content**	
**Beginning**	7E	
**Size**	01 03	
**Transmission method**	01	
**RSSI**	40	
**Reserved**	01	
**Origin address**	C0 A8 05 02	
**Destination address**	C0 A8 04 01	
**Useful charge**	**Type**	44
	**Initial Origin Address**	00 56 45 52
	**Final Destination Address**	00 62 67 62
	**Jumps**	02
	**Source Packet counter**	00 00 0A
	**Data**	“On-Road_Vehicle_Data_Message”:[ { “acknowledgement” : “1”, “latitude” : “19.2651047871”, “temperature” : “26.5”, “vehicleId”: “642c5dd1163518942a44440a145fb1ba5f96787c”, “packageCounter”:”322” “speed” : “0.037”, “timestamp”:“2016-03-28 20:13:42”, “longitude” : “−103.713618619”, }, { “acknowledgement” : “1”, “latitude” : “19.2651047549”, “temperature” : “26.6”, “vehicleId”: “645864b8e49bb0ac130d26de690231f9a9a9069a”, “packageCounter”:”452” “speed” : “0.018”, “timestamp” : “2016-03-28 20:13:42”, “longitude” : “−103.713618561”, }, { “acknowledgement” : “1”, “latitude” : “19.2651047388”, “temperature” : “26.9”, “vehicleId”: “649164b8e49cc0ac130d26de690231f9a9a9879b”, “packageCounter”:”452” “speed” : “0.020”, “timestamp” : “2016-03-28 20:13:42”, “longitude” : “−103.713618532”, }]
	**Latitude**	19.2651047871
	**Longitude**	−103.713618619
**Verification sum**	9D	
**Forcer**	7D	

**Table 9:** Results of precision of the traffic detecting algorithm.

Qty. Vehicles with OBU.	Scheduled Traffic Congestions	Traffic Alerts Generated	False Traffic Alerts Generated	Traffic Alerts not Generated	Percentage of Precision
24 (10.1%)	64	63	1	2	93.7%
118 (50%)	64	63	0	1	98.4%
